# Stable perceptual phenotype of the magnitude of history biases even in the face of global task complexity

**DOI:** 10.1167/jov.23.8.4

**Published:** 2023-08-02

**Authors:** Darinka Trübutschek, Lucia Melloni

**Affiliations:** 1 Research Group Neural Circuits, Consciousness and Cognition, Max Planck Institute for Empirical Aesthetics, Frankfurt/Main, Germany; 2 Department of Neurology, NYU Grossman School of Medicine, New York, New York, USA

**Keywords:** serial dependence, stimulus-history effects, history biases, interindividual variability, intraindividual stability

## Abstract

According to a Bayesian framework, visual perception requires active interpretation of noisy sensory signals in light of prior information. One such mechanism, serial dependence, is thought to promote perceptual stability by assimilating current percepts with recent stimulus history. Combining a delayed orientation-adjustment paradigm with predictable (study 1) or unpredictable (study 2) task structure, we test two key predictions of this account in a novel context: first, that serial dependence should persist even in variable environments, and, second, that, within a given observer and context, this behavioral bias should be stable from one occasion to the next. Relying on data of 41 human volunteers and two separate experimental sessions, we confirm both hypotheses. Group-level, attractive serial dependence remained strong even in the face of volatile settings with multiple, unpredictable types of tasks, and, despite considerable interindividual variability, within-subject patterns of attractive and repulsive stimulus-history biases were highly stable from one experimental session to the next. In line with the hypothesized functional role of serial dependence, we propose that, together with previous work, our findings suggest the existence of a more general individual-specific fingerprint with which the past shapes current perception. Congruent with the Bayesian account, interindividual differences may then result from differential weighting of sensory evidence and prior information.

## Introduction

According to the Bayesian framework, visual perception is an active process (Friston, 2005; “Helmholtz's Treatise on Physiological Optics,” Translated from the Third German Edition 1925; Rao & Ballard, 1999). Whereas our external world tends to be stable over short periods of time, the corresponding sensory brain signals are noisy. An efficient way for our brain to overcome such irrelevant, moment-to-moment fluctuations in noise would thus be to predict the nature of new input based on prior knowledge and information (Clark, 2013; Fecteau & Munoz, 2003; Schwartz, Hsu, & Dayan, 2007; Trapp, Pascucci, & Chelazzi, 2021), thereby promoting perceptual stability and continuity (Fischer & Whitney, 2014; Kiyonaga, Scimeca, Bliss, & Whitney, 2017; Kleinschmidt, Büchel, Hutton, Friston, & Frackowiak, 2002; Snyder, Schwiedrzik, Vitela, & Melloni, 2015).

One potential mechanism, by which the brain may accomplish this feat, is to bias processing of current sensory input in light of recent sensory experience. Such stimulus-history biases have, indeed, been researched and documented for decades: In perceptual hysteresis, an observer's perception of an ambiguous, multi-stable, or weak stimulus on the current trial tends to be attracted toward his or her experience on the previous trial (Gepshtein & Kubovy, 2005; Kleinschmidt et al., 2002; Pearson & Brascamp, 2008; Schwiedrzik, Ruff, Lazar, Leitner, Singer, & Melloni, 2014; Schwiedrzik, Sudmann, Thesen, Wang, Groppe et al., 2018; Sekuler, 1996). For instance, if, at moment *n*-1, an individual perceived the duck in the famous duck-rabbit drawing, he or she will likely continue to experience the duck at moment *n* as well. Such perceptual stabilization based on previous stimuli has recently also been extended to the context of un-ambiguous, clearly visible stimuli – a phenomenon termed serial dependence (Fischer & Whitney, 2014).

If serial dependence and related stimulus-history biases are indeed a general-purpose mechanism of the visual system to stabilize perception, then a number of different conditions should be met. First, within to-be-established boundaries, they should be observed for a wide variety of stimulus features, stimulus categories, and, importantly, experimental tasks. Second, they should occur not only at the group-level, but also at the level of individual observers, shaping perception in most (if not, all) individuals. Third, and, perhaps most importantly, there should also exist a unique stable perceptual phenotype, such that for a given individual and context, across several occasions, stimulus history biases current perception with a given magnitude and/or tuning.

Over the past decade, empirical evidence supporting all three prerequisites has started to accrue. Apart from stimulus orientation (Cicchini, Benedetto, & Burr, 2021; Fischer & Whitney, 2014; Fritsche, Mostert, & de Lange, 2017; Murai & Whitney, 2021; Pascucci, Mancuso, Santandrea, Della Libera, Plomp, & Chelazzi, 2019), serial dependence has already been reported for many other low- and high-level stimulus features, including color (Barbosa & Compte, 2020), luminance (Frund, Wichmann, & Macke, 2014), motion direction (Alais, Leung, & Van Der Burg, 2017; Czoschke, Fischer, Beitner, Kaiser, & Bledowski, 2019; Fischer, Czoschke, Peters, Rahm, & Bledowski, 2020), spatial location (Bliss, Sun, & D'Esposito, 2017; Manassi, Liberman, Kosovicheva, Zhang, & Whitney, 2018), attractiveness judgments (Kok, Taubert, Van Der Burg, Rhodes, & Alais, 2017; Van der Burg, Rhodes, & Alais, 2019; Xia, Leib, & Whitney, 2016), facial identity (Liberman, Fischer, & Whitney, 2014; Taubert, Alais, & Burr, 2016), and numerosity (Cicchini, Anobile, & Burr, 2014; Corbett, Fischer, & Whitney, 2011; Fornaciai & Park, 2018).

Although most of this work has presented group-level results, there is also increasing interest in assessing the way in which recent experience shapes the perception and behavior of individual observers. Serial dependence cannot only be reliably measured at the single-subject level (Fischer & Whitney, 2014; Manassi, Murai, & Whitney, 2018), but has also been found to vary considerably between different individuals. For instance, Bliss and colleagues (2017) observed that, in a standard spatial delayed response task, only approximately half of the participants displayed the attractive effect typical of group-level serial dependence, with the remainder either showing an effect in the opposite direction (i.e. repulsion) or no clear bias at all. As stipulated by the Bayesian framework of vision as active inference (Zhang & Alais, 2020), such interindividual differences in the magnitude and/or direction of stimulus-history biases could arise from differences in precision weighting of sensory evidence and prior knowledge. Indeed, individual differences in serial dependence seem particularly pronounced when sensory uncertainty is high (Kim & Alais, 2021), and there appear to be interobserver differences in weighting of prior stimuli versus prior responses (Zhang & Alais, 2020).

Although, so far, much less explored, intriguing recent work by two groups suggests that, despite these considerable interindividual differences in serial dependence, there could, in fact, be a stable perceptual phenotype. Kondo, Murai, and Whitney (2022), on one hand, assessed test-retest reliability of serial dependence magnitude using a typical delayed orientation reproduction task, with stimuli either presented in the same or different spatial positions on two occasions. Although observers who were tested on the same spatial locations on both days displayed similar serial dependence in both sessions, no such pattern was observed if spatial location of the stimuli changed between sessions. On the other hand (Van Geert, Moors, Haaf, & Wagemans, 2022) relied on ambiguous dot-lattice stimuli to demonstrate that individual differences in both attractive and repulsive history biases remain stable for a period of up to 2 weeks.

Whereas, together, the available evidence is consistent with serial dependence being a general-purpose mechanism of the visual system with a subject-specific fingerprint, a critical aspect has, so far, remained largely unexplored: the structure, predictability, and complexity of the experimental task. With few exceptions (Cicchini, Mikellidou, & Burr, 2017), previous work has used highly structured and predictable experimental settings, in which observers repeatedly perform the exact same judgment on every trial. Yet, there is some evidence to suggest that the strength of group-level serial dependence may be modulated by the local structure of the environment, for instance, being reduced at transitions toward a new perceptual context (Fischer et al., 2020; Kiyonaga, Manassi, D'Esposito, & Whitney, 2017). The global structure and complexity of an experimental task could potentially have an even stronger effect: If the task to be performed with a given object varies in a predictable or unpredictable fashion, it is conceivable that, even during episodes of relative task stability in such a variable, volatile environment, reliance on the recent past to guide current perception and behavior is reduced or even entirely erased. This could either abolish group-level serial dependence (e.g. due to increased interobserver variability) or specifically affect the stability with which stimulus history biases current perception on different occasions in individual observers (thereby casting doubt on the existence of a general, subject-specific phenotype).

We here set out to provide a first insight into this open issue by testing for the existence of group-level and single-subject serial dependence in the context of a predictable (study 1) or unpredictable (study 2) global experimental task structure. Note that, for the intents and purposes of the current study, we focused exclusively on establishing whether or not serial dependence exists in such volatile environments, rather than on interrogating the role of differences in degree of predictability on stimulus history biases (which will be the subject of future reports). In addition, as a second main objective, we also aimed at exploring whether the recently reported pattern of within-observer stability of serial dependence and related mechanisms would also hold in such variable task environments.

## Materials and methods

### Participants

We included a total of 41 healthy adult volunteers (26 women and 15 men; *M*_age_ ± standard deviation [*SD*] = 30.88 ± 8.24 years) in the current analyses: 21 participants (12 women and 9 men; *M*_age_ ± *SD* = 28.62 ± 8.38 years) from study 1 and 20 participants (14 women and 6 men; *M*_age_ ± *SD* = 33.25 ± 7.39 years) from study 2. All reported normal or corrected-to-normal vision. Each participant took part in two experimental sessions, spaced between 1 and 30 days apart (*M* ± *SD* = 4.02 ± 6.07 days), and lasting between 2.5 and 3 hours each. Participants received a compensation of 70€, and provided written informed consent at the beginning of the first session. Experimental procedures abided by the guidelines of the Declaration of Helsinki and had been approved by the Ethics Committee of the Max Planck Society.

### Stimulus presentation procedures

We programmed our experiments in PsychoPy version 2021.2.3 (Peirce, Gray, Simpson, MacAskill, Höchenberger, et al., 2019), run in an Anaconda environment. Experimental tasks were displayed on a gray background (red, green, and blue [RGB] = [125, 125, 125]; 26.34 cd/m^2^) on a BenQ XL24020Z 24-inch monitor, (1920 × 1080 pixels, 120 Hertz [Hz]) connected to a 64-bit FUJITSU computer running Windows 10. The experiments were performed in a soundproofed and dimly lit experimental booth with tightly controlled ambient light conditions, held constant across experimental sessions and individual observers. Participants sat at a distance of approximately 90 cm from the computer screen, with their head positioned on a chin rest. They were instructed to maintain their gaze at the center of the screen for the entire duration of the experiments.

### Basic features of the delayed orientation adjustment task

We adapted the classic serial dependence paradigm (Fischer & Whitney, 2014) to (1) interrogate the existence of stimulus-history effects in the context of global task complexity and (2) further test the possibility of a stable observer-specific fingerprint with which stimulus history affects current perception and behavior. Although the strength of group-level serial dependence appears to be larger under unpredictable (i.e. study 2) versus predictable (i.e. study 1; cf. below) task structure, please note that a thorough investigation of this aspect of the data is the topic of future reports.

**Figure 1.**
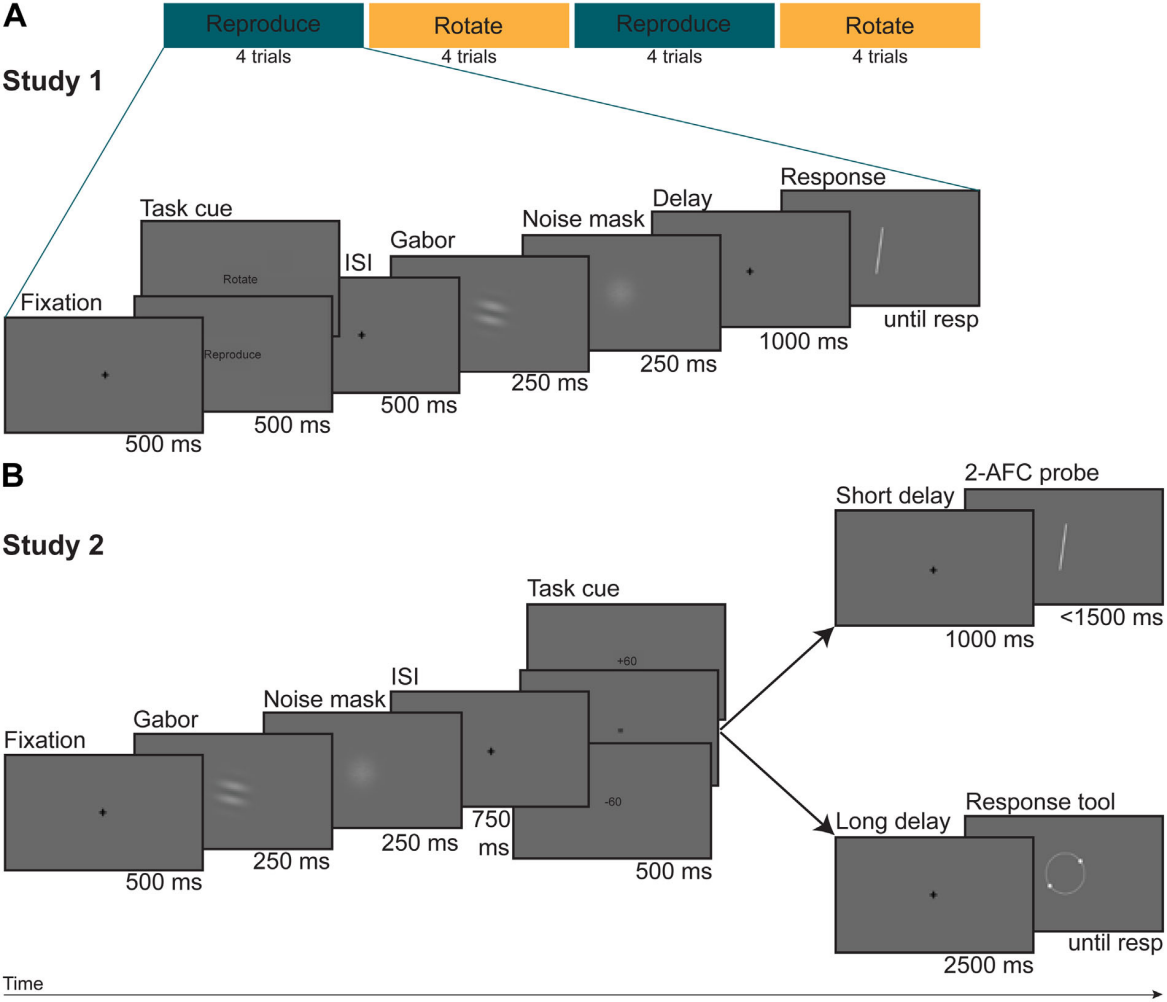
Experimental paradigms. (**A**) Schematic of experimental procedure in study 1. On each trial, a task cue instructed subjects as to the specific task to be performed at the end of the trial. In case of no-rotation trials (signaled by the cue “Reproduce”), they were to reproduce the orientation of a Gabor stimulus by rotating the response bar until its tilt matched the orientation of the Gabor. In case of rotation trials (signaled by the cue “Rotate”), they were to mentally rotate the Gabor orientation by either 60 degrees clockwise or counterclockwise and then reproduce that rotated orientation. Task sequence (i.e. no-rotation vs. rotation) was predictable, with task switches occurring after every 4 trials. Rotation direction (i.e. clockwise vs. counterclockwise) was constant within a given experimental session (counterbalanced across participants). On 20% of all trials, the response bar was replaced with a blank screen (with fixation), shown for the average duration of a subject's previous reaction times. In the current analyses, we only focused on no-rotation trials with a response preceded by at least another no-rotation trial requiring a response. (**B**) Schematic of experimental procedure in study 2. On each trial, subjects again had to either reproduce (signaled by =) or rotate by 60 degrees clockwise (signaled by +60) or 60 degrees counterclockwise (signaled by −60) the orientation of a Gabor patch. In contrast to study 1, the task cue appeared after the presentation of the Gabor stimulus (i.e., post-cue), task sequence (i.e. no-rotation vs. rotation) was unpredictable, changing randomly from trial to trial, and both rotation directions could occur within a given experimental session. Moreover, we replaced the response bar with a response tool in order to minimize the effects of additional sensory orientation signals on stimulus-evoked serial dependence and, on a subset of 27.27% of all trials replaced the delayed adjustment task with a speeded two-alternative forced-choice (2-AFC) task in order to encourage mental rotation of the Gabor orientation. In this task, a tilted line either matched (i.e. delta angle = 0 degrees) or did not match (i.e. delta angle = +/− 30 degrees) the expected correct response orientation of the Gabor, and participants had to decide which of these two alternatives was the case. Again, in the current analyses, we only focused on adjustment no-rotation trials preceded by at least one other adjustment no-rotation trial. ISI = inter-stimulus interval, resp = response.

As can be seen in Figure 1, randomly oriented Gabor patches (i.e. windowed sine wave gratings; 5 degrees of visual angle [dva]) were shown for 250 ms at the center of the computer screen. They had a Michelson contrast of 30%, a spatial frequency of 0.75 cycles/degree, and were windowed with a Gaussian envelope. We not only followed Fritsche and colleagues’ example (Fritsche et al., 2017; Fritsche, Spaak, & de Lange, 2020) and presented Gabor gratings at a fixed phase, but in addition, in line with some previous studies (Kondo et al., 2022; Murai & Whitney, 2021; Pascucci et al., 2019), showed them foveally (rather than peripherally). Whereas this was necessary to simplify an already challenging experimental task, this choice increased sensory reliability of the stimuli, thereby potentially affecting our ability to detect subtle serial dependence effects (Cicchini, Mikellidou, & Burr, 2018; Gallagher & Benton, 2022). Immediately afterward, a noise mask (i.e. white noise patch of the same size as the Gabor stimulus and smoothed with a Gaussian kernel with an *SD* of 2 degrees) replaced the Gabor for 250 ms to minimize negative orientation aftereffects. Although not rigorously equating between mean luminance of the noise patch and mean luminance of the screen's background carries the risk of generating negative luminance aftereffects, these would have been weak, short-lived, and, importantly, unrelated to the orientation effects under investigation here.

Following a 1 second (study 1) or 2.5 seconds-long (study 2) delay period with fixation, participants reproduced the Gabor orientation by adjusting a random orientation. In study 1, on 80% of the trials, a white response bar (0.2 dva wide, 2 dva high, and 58.20 cd/m^2^), smoothed with a Gaussian kernel, was presented at the same location as the preceding stimuli. The remaining 20% of the trials, pseudo-randomly interspersed in the trial sequence, served as a no-response control condition, in which the response bar was replaced with a fixation cross (shown for the average duration of a participant's previous responses) and participants were instructed to wait for the beginning of the next trial. By contrast, in study 2, a response tool (RGB = [1, 1, 1], 58.20 cd/m^2^, 1.75 dva; see Figure 1B), forming an imaginary line and intended to minimize the effect of additional sensory orientation signals on stimulus-evoked serial dependence (Ceylan, Herzog, & Pascucci, 2021), appeared at the same location as the preceding Gabor patch.

A given trial ended with a fixation cross either randomly drawn from a uniform distribution between 1300 and 1700 ms (study 1) or fixed at 600 ms (study 2). In study 2, the color of the fixation cross provided immediate feedback about participants’ accuracy in the adjustment task (green = <10 degrees deviation, orange = 10 to <15 degrees deviation, and red = >15 degrees deviation). There was a short break every 72 (study 1) or 60 (study 2) trials, at which time participants received feedback about their mean absolute response error.

### Manipulating global task complexity by introducing predictable (study 1) or unpredictable (study 2) changes in task

Previous reports of serial dependence have primarily been based on experimental tasks in which every trial required the same type of response. Here, we increased task complexity by varying the required response in a predictable (study 1) or unpredictable fashion (study 2). Participants reported the actual Gabor orientation (no-rotation trials) or an orientation that was rotated 60 degrees clockwise or counterclockwise (rotation trials). Predictability of the tasks varied between the two experiments.

While augmenting task complexity with respect to previous work on serial dependence, in study 1, we wanted to otherwise minimize noise as much as possible. Task instructions were displayed in the form of a pre-cue (i.e. reproduce vs. rotate; RGB = [1, 1, 1], 58.20 cd/m^2^, 1 dva; 500 ms) 500 ms prior to the Gabor, the temporal sequence of the task to be performed on each trial (i.e. no-rotation vs. rotation) followed a predictable pattern (i.e. AAAA BBBB AAAA, etc.), and rotation direction was kept constant within a given experimental session (counterbalanced across participants).

By contrast, in study 2, the task cue (i.e. −60 [counterclockwise rotation], = [no rotation], +60 [clockwise rotation]; RGB = [1, 1, 1], 58.20 cd/m^2^; 1 dva; 500 ms) was post-cued 750 ms after the offset of the noise mask, and task order and rotation direction varied randomly from trial to trial.

### Encouraging the performance of mental rotations prior to response selection

In study 2, we incorporated further measures to ensure that, on rotation trials, participants performed the rotation task mentally – prior to adjusting the response tool.

First, we preselected participants based on an online pretest using a modified version of the standard Mental Rotation Test (Peters, Laeng, Latham, Jackson, Zaiyouna, & Richardson, 1995; Peters & Battista, 2008).

Second, on a random subset of 27.27% of all trials, we replaced the delayed orientation adjustment task with a two-alternative forced-choice (2AFC) task. Here, 1 second after the offset of the task cue, a tilted line (RGB = [1, 1, 1], 58.20 cd/m^2^, 2 dva) was shown for a maximum of 1.5 seconds. This probe either matched the expected correct response orientation (in 50% of the cases), or it was tilted 30 degrees away from it in the clockwise or counterclockwise direction. Participants gave a speeded response as to whether or not the orientation of the probe matched the orientation they currently held in mind. This 2AFC task occurred more frequently on rotation than on no-rotation trials (i.e. 66.6% vs. 33.3%) and encouraged participants to adopt a strategy, in which they completed the rotation mentally during the delay period of the adjustment task. For any analysis, we only consider participants whose accuracy on the 2AFC task exceeds 65%.

### Overall task structure

We counterbalanced angular distances between orientations on trial *n* and trial *n*-1 with respect to serial position (i.e., 1–4) within a given miniblock in study 1 and, similarly, with respect to task (i.e. no-rotation vs. rotation) in study 2. We first divided the distance space from −90 degrees to +90 degrees into 18 equally spaced bins, and then distributed those equally among the four different serial positions or among no-rotation and rotation trials as well as clockwise and counterclockwise rotation directions. Actual angular orientations on each trial were determined by randomly choosing an initial angle for the first trial and applying the sequence of operations as defined by the distance bins. This counterbalancing ensured that, over the course of the experiments, each distance bin was repeated 32 times/serial position (i.e. 128 times/task) in study 1 and 40 times/task on no-2AFC trials in study 2.

### Experimental protocol

To assess within-subject stability of stimulus-history effects, participants completed two experimental sessions. Each lasted between approximately 2.5 to 3 hours and took place on separate days. At the beginning of each session, participants completed 60 training trials, identical to the main task (except that visual feedback was provided at the end of each trial) in study 1. In study 2, they performed three training tasks at the beginning of the first session: training task 1 allowed participants to practice a mental rotation of 60 degrees; training task 2 familiarized participants with the 2AFC task; and training task 3 consisted of 60 trials identical to the main task. Participants then completed eight experimental blocks à 144 trials in each session of study 1 (i.e. 2304 trials total), and two experimental blocks à 396 trials in session 1 of study 2, followed by three experimental blocks in session 2 (i.e. 1980 trials total).

### Data preprocessing

To aggregate data from the two studies, we exclusively focus on no-switch, no-rotation trials. From study 1, we included 10,080 no-rotation trials that required a response and were preceded by another response no-rotation trial (i.e. 480 trials/participant). Similarly, from study 2, we included 5145 no-rotation trials that required an adjustment response and were preceded by the same trial category (i.e. *M* ± *SD* = 257.25 ± 10.23 trial/participant).

We followed standard procedures to preprocess our data for the serial dependence analysis (Fritsche et al., 2017). First, to capture potential lapses in attention, for each participant and session, we removed trials, in which participants’ response error (i.e. shortest angular distance between the correct response orientation and observed response orientation) differed by more than three circular *SDs* from a participant's session-wide mean response error (study 1 = 4.75 ± 4.15 [*M* ± *SD*] trials/participant; study 2 = 1.93 ± 2.17 [*M* ± *SD*] trials/participant). Next, again separately for each participant and session, we extracted residual response errors by computing overall bias (i.e. mean directional response error) and then subtracting this from each individual response error. It is this bias-free residual response error that we consider in the subsequent serial dependence analyses.

### Quantifying serial dependence using model-based analysis

To quantify systematic history biases, we expressed participants’ residual response errors (i.e. observed response orientation – correct response orientation; y-axis) on the current trial as a function of angular distance between stimulus orientation on the current and the previous trial (i.e. previous – current stimulus orientation; x-axis). Positive values signal a clockwise displacement of the response or previous stimulus orientation, whereas negative values indicate a counterclockwise displacement. If, for a given trial, both the response error and the stimulus distance had the same sign, they tended in the same direction and, as such, would constitute an attractive serial dependence effect. Opposite signs for response errors and stimulus distance, by contrast, signal a repulsive adaptation effect of the past.

In keeping with the most recent literature (Kondo et al., 2022; Manassi, Ghirardo, Canas-Bajo, Ren, Prinzmetal & Whitney, 2021), we fit a derivative-of-von-Mises function (i.e. DvM) to the resulting patterns of pooled or moving-average (window size of 20 degrees) response errors for group- and subject-level analyses, respectively. In contrast to the more widely used derivative-of-Gaussian (i.e. DoG) function (Fischer et al., 2020; Fischer & Whitney, 2014; Fritsche et al., 2020), the periodic DvM is specifically adapted to a circular space. Given by

$$\begin{array}{c} y=-\frac{a\kappa \sin\left(x-\mu \right)e^{\kappa \;\cos\left(x-\mu \right)}}{2\pi I_{0}\left(\kappa \right)}, \end{array}$$

here, *y* denotes the response error on each trial, *x* the relative orientation on the previous trial, *a* the amplitude of the curve's peak, *κ* the concentration of the DvM, µ the function's symmetry axis, and *I*_0_(*κ*) the modified Bessel function of order 0. Although we fixed µ to 0, we treated *a* and *κ* as free parameters, constraining them to a wide range of plausible values (i.e. *a* = [−15, 15] and *κ* = [0, 200]). Half the peak-to-trough amplitude of the resulting model fit is our measure of the strength (or magnitude) of stimulus-history effects, whereas its full width at half maximum (FWHM) serves as our measure of the tuning of serial dependence. Please note that, in cases in which the model fit was shallow and, as a result, the FWHM would have exceeded our bound of 0 degrees and 90 degrees, the FWHM was not computed. To obtain estimates of the variability of the best-fitting model parameters for the group-level analyses, we computed bootstrapped distributions by fitting the DvM to 1000 resamples of our data (done with replacement) and computing the bootstrapped distribution's *SD*.

### Assessing statistical significance of serial dependence

We relied on permutation tests to assess statistical significance at the group- and the single-subject level. Any dependence between response errors and stimulus history was removed by shuffling the sequence of each observer's angular distances. We then refit the DvM to these shuffled data 1000 times, storing all parameters of the best-fitting DvM curve on each iteration. Statistical significance was determined by comparing half the peak-to-trough amplitude derived from fitting the actual data against the corresponding permutation distribution. We derived one-sided (at the group-level, where positive serial dependence is expected) or two-sided (at the single-subject level, where both attractive and repulsive effects may exist) *p* values by computing the percentage of permutations that led to equal or higher and/or lower values for half the peak-to-trough amplitude than the one estimated on the empirical data.

### Quantifying serial dependence using model-free analysis

As has been noted before (Fritsche et al., 2017; Gallagher & Benton, 2022; Samaha, Switzky, & Postle, 2019), quantifying serial dependence based on model-fitting oftentimes results in spurious fits and, hence, meaningless estimates of serial dependence. To overcome this issue, we additionally adopted a model-free approach to quantify the magnitude of serial dependence. Following previous work (Fritsche et al., 2017; Samaha et al., 2019), we first binned response errors according to whether, within a given range, the stimulus orientation on the previous trial was counterclockwise or clockwise to the orientation on the current trial and then calculated the circular mean for each of these bins. We subtracted the former from the latter, resulting in a positive value when, on average, responses in the two bins are attracted toward the previous stimulus orientation, and a negative value when, on average, responses are biased away. We chose the specific bounds of the angular distance ranges to be included to be −54 and 54 in study 1 and −86 and 86 in study 2, corresponding to 99.8% of the area under the right (i.e. positive) curve of the pooled group data (see Figure 3). Statistical significance of model-free estimates of serial dependence was obtained by comparing the empirically derived bias estimate to a permuted null distribution (built by shuffling bin category).

## Results

### Objective task performance is comparable across studies

**Figure 2.**
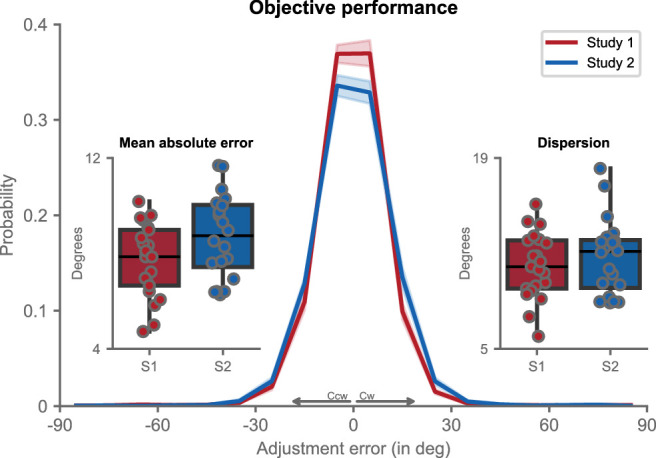
Objective performance. Adjustment error (i.e. response orientation - stimulus orientation) distributions are shown separately for study 1 (in *red*) and study 2 (in *blue*). Positive errors denote a clockwise displacement of the response orientation with respect to the stimulus orientation, negative errors a counterclockwise displacement. *Thick lines* represent group mean, and the *shaded area* denotes standard error of the mean (SEM). Boxplots show mean absolute error (*left*) and dispersion (i.e. circular standard deviation; right). Within each boxplot, the *horizontal black line* is the group median and whiskers represent 1.5 interquartile ranges (IQRs) of the lower and upper quartile. Each scatter corresponds to a subject. S1 = study 1, S2 = study 2.

We first compared participants’ performance on the adjustment task across the two studies. Distributions of response errors were centered on 0 (Figure 2), confirming that, despite task difficulty, participants reproduced the Gabor orientation with high accuracy. Mean absolute response error was 7.71 degrees ± 0.34 degrees (*M* ± *SE*) in study 1 and relatively similar in study 2 (8.71 ± 0.39 degrees; *F*(1, 39) = 3.82, *p* = 0.058, *η^2^* = 0.089), with both being comparable in magnitude to previous reports (Fritsche et al., 2017). Dispersion (i.e. circular standard deviation) of the response error distributions was equally small in both studies (study 1: *M* ± *SE =* 11.08 degrees ± 0.53 degrees; study 2: *M* ± *SE =* 11.76 degrees ± 0.63 degrees; *F*(1, 39) = 0.69, *p* = 0.411, *η^2^* = 0.017). Objective task performance was thus highly similar across the two studies – both in terms of accuracy and precision.

### Serial dependence effect at the group-level persists even for complex tasks

The vast majority of previous work has examined the existence and characteristics of serial dependence in the context of experimental scenarios with a single and/or predictable task. Will stimulus history bias current perception and behavior even when the predictive value of the recent past may be diminished due to predictable (study 1) or unpredictable (study 2) task changes?

**Figure 3.**
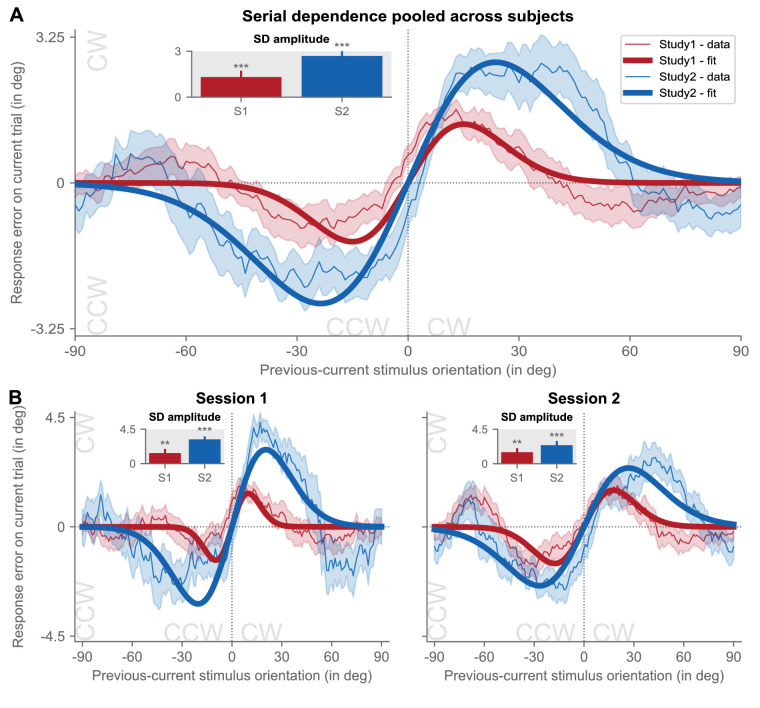
Classic serial dependence at the group-level. (**A**) Group-level serial dependence across experimental sessions. Response errors on the adjustment task (i.e. response orientation - stimulus orientation; y-axis) on the current trial are shown as a function of relative angular distances between previous and current stimulus orientations (x-axis) for study 1 (*red*) and study 2 (*blue*). For positive y-values, the current response error was in the clockwise direction, and for positive x-values, the previous stimulus was oriented more clockwise than the current stimulus. As revealed by the smoothed (for visualization purposes only) mean response errors (thin colored lines with standard error [SEM] shown as the *shaded area*), responses were systematically biased toward the previous stimulus. This bias followed a derivative-of-von-Mises (DvM) shape, with model fits shown as *bold colored lines*. Solid lines indicate a significant fit at an alpha-level of *p* < 0.05 as assessed by comparing actual half peak-to-trough amplitude against a permuted null-distribution of amplitudes. We take the amplitude of models with significant fits as a proxy for the strength of serial dependence and plot these, alongside the bootstrapped standard deviations, as an inset. *Asterisks* above bar plots in the inset denote statistical significance as derived from permutation testing of amplitudes. * *p* < 0.05, ** *p* < 0.01, *** *p* < 0.001. S1 = study 1, S2 = study 2, SD = serial dependence. (**B**) Same as in panel **A**, but for group-level serial dependence in session 1. (**C**) Same as in panel **A**, but for group-level serial dependence in session 2.

In order to address this first question of the current analyses, we plotted participants’ pooled adjustment errors as a function of relative stimulus orientation on the current trial (see Figure 3). In both studies, we observed classic attractive serial dependence, with response errors following a DvM shape (see Figure 3A).

This attraction was numerically more pronounced in study 2 than in study 1 (amplitude ± bootstrapped *SDs* = 2.69 degrees ± 0.45 degrees vs. 1.31 degrees ± 0.41 degrees; FWHM = 38.78 degrees vs. 24.35 degrees; *R^2^* = 0.021 vs. 0.004), perhaps due to differences in the durations of the delay period, but statistically still highly significant in both studies (all *p* values < 0.001).

Critically, angular distance between the orientations of the current and the upcoming Gabor stimulus did not systematically modulate response errors (study 1 vs. study 2: amplitude ± bootstrapped *SDs* = 0.51 degrees ± 0.52 degrees vs. −0.23 degrees ± 0.64 degrees; FWHM = 6.47 degrees vs. 70.55 degrees; *R^2^* = 0.0002 vs. 0.0003; *p* = 0.072 vs. 0.569; Supplementary Figure S1), confirming that the attractive bias we observed with respect to previous stimuli did not arise due to statistical regularities in participants’ stimulus sequences.

This group-level serial dependence remained strong even when considering session 1 (see Figure 3B) and session 2 (see Figure 3C) separately (session 1 - study 1 vs. study 2: amplitude ± bootstrapped *SDs* = 1.37 degrees ± 0.55 degrees vs. 3.17 degrees ± 0.38 degrees; FWHM = 15.70 degrees vs. 33.28 degrees; *R^2^* = 0.002 vs. 0.022; *p* = 0.009 vs. 0.001; session 2 - study 1 vs. study 2 = amplitude ± bootstrapped *SDs* = 1.51 degrees ± 0.52 degrees vs. 2.42 degrees ± 0.55 degrees; FWHM = 28.62 degrees vs. 44.19 degrees; *R^2^* = 0.007 vs. 0.021; *p* = 0.003 vs. 0.001). Importantly, there was no clear positive relationship between the number of trials included in the model fits and either magnitude of estimated serial dependence amplitude (Supplementary Figure S2A) or quality of model fit (Supplementary Figure S2B).

As such, these findings demonstrate that classic serial dependence assessed at the group-level exists even in the context of a globally variable task structure, and that the quality and strength of this attraction does not scale with sample size.

### The effect of stimulus history on current perception is variable between individual observers

**Figure 4.**
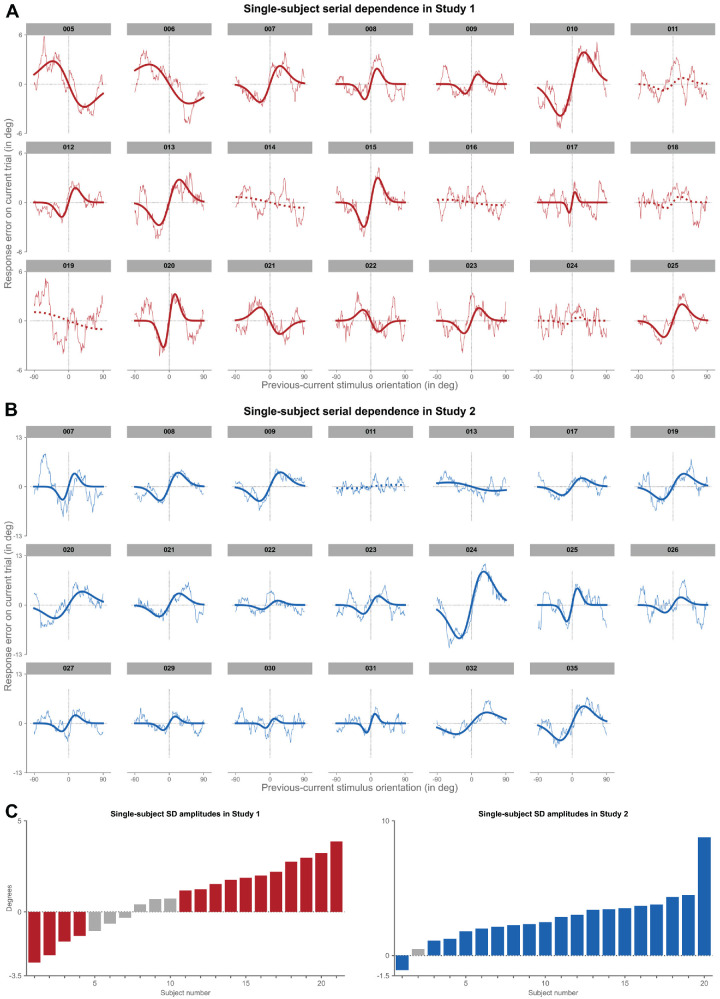
Pattern of behavioral bias due to stimulus-history is highly variable between-subjects. (**A**) Single-subject serial dependence in study 1. Each graph depicts moving-average, single-subject response errors (*thin line*) on the adjustment task (i.e. response orientation – stimulus orientation; y-axis) on the current trial as a function of relative angular distances between previous and current stimulus orientations (x-axis). For positive y-values, the current response error was in the clockwise direction, and for positive x-values, the previous stimulus was oriented more clockwise than the current stimulus. We fit a derivative-of-von-Mises (DvM) function to each subject's data and assessed statistical significance by comparing actual half peak-to-trough amplitude against a permuted null-distribution of amplitudes. *Solid lines* indicate model fits with significant serial dependence at an alpha-level of *p* < 0.05 and *dotted lines* represent nonsignificant model fits. (**B**) Same as in panel **A**, but for study 2. (**C**) Sorted magnitudes of serial dependence observed for individual subjects in study 1 (*left*) and study 2 (*right*). Each bar plot shows the half peak-to-trough amplitude of the best-fitting DvM model of a given subject. Subjects with significant fits (i.e. *p* < 0.05) are colored in *dark red* (study 1) or *dark blue* (study 2). Note that the order of subjects does not correspond to the order of subjects in panels **A** or **B**. SD = serial dependence.

So far, we have extended previous reports of serial dependence by showing that, at the group-level, even in complex task environments of variable predictability, stimulus history shapes current perception and behavior. However, whether such group-level results may also be observed at the level of individual observers and, if so, how representative they may be is not yet clear. To quantify interindividual differences in serial dependence in such complex experimental settings, we fit the DvM-function separately to each participant in the two studies (Figure 4).

Critically, in this initial analysis, we collapsed data from both sessions to increase available trial numbers per participant. Similar to previous reports (Bliss et al., 2017), even under these variable task conditions, the vast majority of participants showed a systematic history bias in study 1 and study 2 (i.e. *p* < 0.05; study 1 vs. study 2: *n*: 15 vs. 19). There were only seven participants in total, six in study 1 and one in study 2, who either showed a very weak variation as a function of stimulus history, or no discernable pattern at all (i.e. *p* > 0.05).

**Figure 5.**
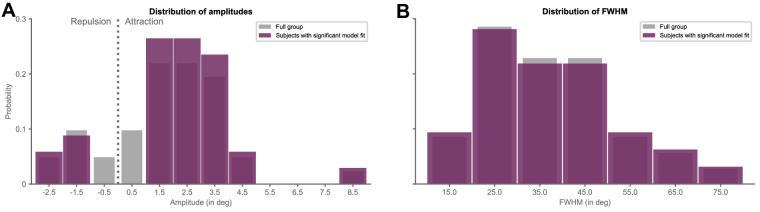
Serial dependence magnitude and width are variable between subjects. (**A**) Histogram of amplitudes obtained from the best-fitting derivative-of-von-Mises (DvM) fits to moving-average individual-subject response errors combined for all subjects across study 1 and study 2 (in *gray*; *n* = 41), and for that subset of subjects with a significant history bias as assessed by comparing actual half peak-to-trough amplitude against a permuted null-distribution of amplitudes (in *purple*; *n* = 34). Labels on the x-axis denote the center of the histogram bins. (**B**) Same as in panel **A**, but for width of serial dependence tuning (i.e. full width at half maximum [FWHM]). Please note that FWHM could only be computed for that subset of subjects (i.e. *n* = 35) with width < 90 degrees.

Critically, however, even within the subset of participants with a clear and significant bias in response errors, there was still considerable variability in the tuning of the effect (see Figures 4, 5). Participants displayed both repulsive and attractive effects, with the magnitudes of serial dependence ranging from −2.78 degrees to 8.78 degrees (*M* ± *SE* = 2.12 degrees ± 0.37 degrees; one-sample *t*-test against 0: *t*(33) = 5.66, *p* < 0.001, Cohen's *d* = 0.97; see Figure 5A). Similarly, the range of stimulus distances over which serial dependence operated varied widely between participants, with some showing rather narrow, and others very broad tuning (i.e. FWHM; range = 12.12 degrees – 70.81 degrees; *M* ± *SE* = 37.60 degrees ± 2.49 degrees; see Figure 5B). There was thus considerable interindividual variation with respect to the existence of stimulus-history effects.

### The magnitude with which stimulus history shapes current perception is stable between two testing sessions within a given observer even in a complex experimental setting

Given this large between-subject variability, we next wondered how stable the patterns of history biases within a given observer would be. If one of the functions of serial dependence is indeed to automatically stabilize perception in the face of noisy sensory input (Fischer & Whitney, 2014), then one might expect that there should be a unique fingerprint of stimulus-history biases for any given observer. Indeed, recent work by two groups (Kondo et al., 2022; Van Geert et al., 2022) has already provided initial evidence for such a stable perceptual phenotype in the context of a standard experimental structure with a single, predictable task. However, whether the observed strong test-retest reliability of the magnitude of serial dependence is a general feature or confined to a limited set of experimental tasks with specific characteristics is still unknown. Moreover, whether this stability only includes the strength with which the recent past modulates current perception and behavior or also extends to its tuning has also not been reported before.

**Figure 6.**
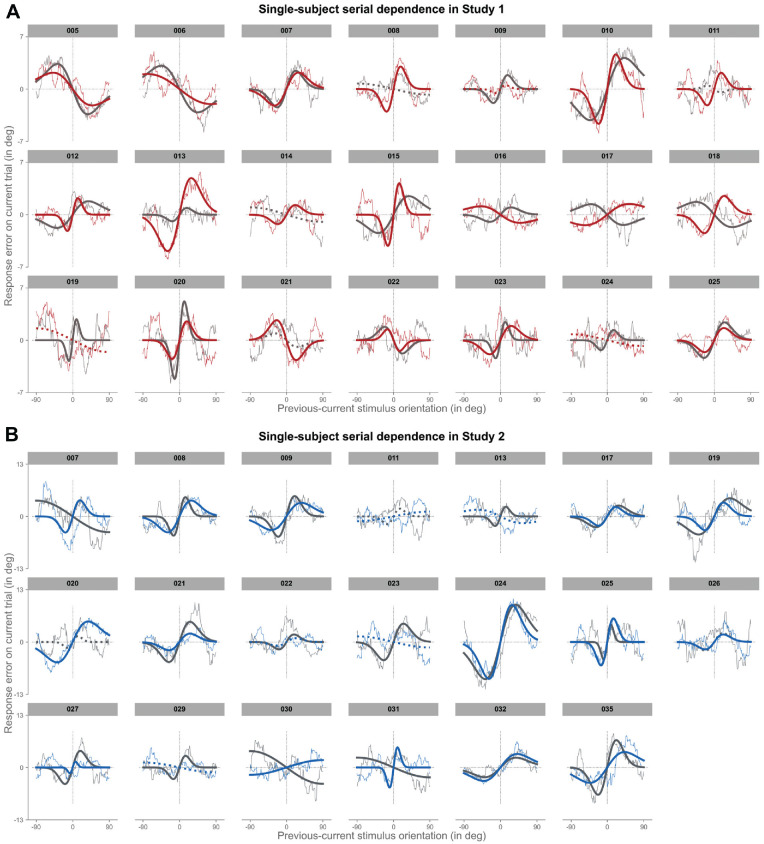
Patterns of behavioral bias due to stimulus-history are highly stable within a given subject. (**A**) Single-subject, single-session serial dependence in study 1. Each graph depicts smoothed single-subject response errors (*thin lines*) on the adjustment task (i.e. response orientation – stimulus orientation; y-axis) on the current trial as a function of relative angular distances between previous and current stimulus orientations (x-axis) as well as experimental session (session 1 = *gray* and session 2 = *colored*). For positive y-values, the current response error was in the clockwise direction, and for positive x-values, the previous stimulus was oriented more clockwise than the current stimulus. We fit a derivative-of-von-Mises (DvM) function to each subject's data and assessed statistical significance by comparing actual half peak-to-trough amplitude against a permuted null-distribution of amplitudes. *Solid lines* indicate model fits with significant serial dependence at an alpha-level of *p* < 0.05 and *dotted lines* represent nonsignificant model fits. (**B**) Same as in panel **A**, but for study 2.

In order to address these questions, we first plotted each participant's response errors as a function of relative distance between the current and previous Gabor stimulus separately for the two experimental sessions, and then fit the DvM-function to all of these curves. The results are remarkable for two reasons (Figure 6): On the one hand, even though noisier due to the reduced number of trials, the vast majority of participants who had displayed a systematic history bias when collapsed across the two sessions (see Figure 4) still displayed significant serial dependence in both of the sessions (i.e. 12 out of 15 participants in study 1, and 13 out of 19 participants in study 2). The remaining participants (i.e. 3 in study 1, and 6 in study 2) showed a clear bias in only one of the two sessions (potentially due to low trial numbers).

On the other hand, and perhaps even more importantly in light of the current questions, the pattern of response bias as well as the resulting model fits appeared remarkably consistent between sessions for individual observers. Indeed, there were only six participants (i.e. subjects 016, 017, and 018 in study 1 and subjects 007, 030, and 031 in study 2), who displayed a reversal of their bias from attractive to repulsive or vice versa. Critically, only four of those seem to have resulted from a genuine reversal, as the other two (i.e. subjects 007 and 031 in study 2) appear to have been driven primarily by spurious model fits.

**Figure 7.**
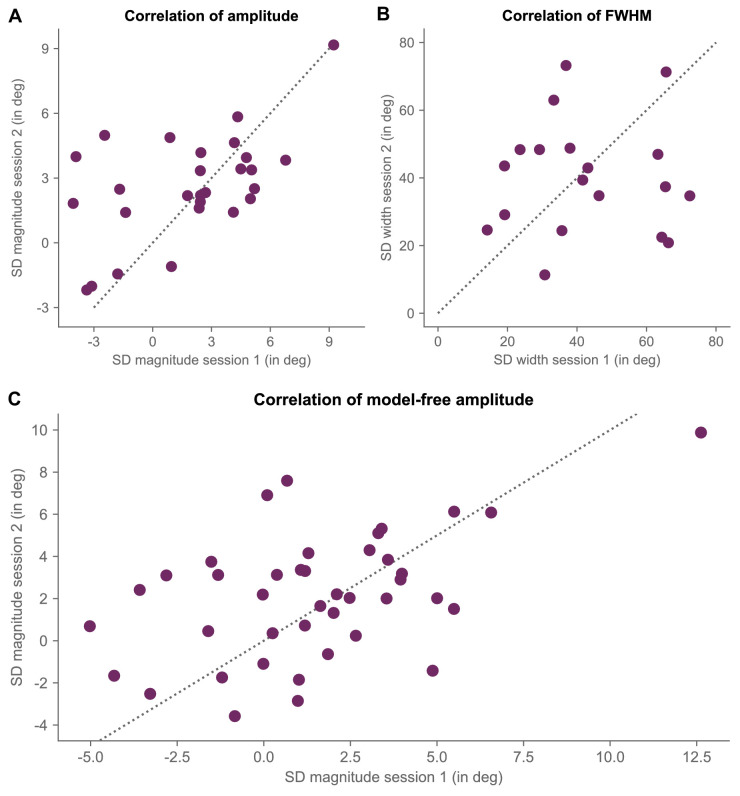
Within-subject stability of serial dependence magnitude and tuning. (**A**) Scatterplot depicting a given subject's serial dependence amplitude for session 1 as a function of the serial dependence amplitude as measured for session 2. Each dot represents the data for a given individual. Note that we only included subjects who displayed significant (i.e. *p* < 0.05) serial dependence for both sessions (as assessed by comparing actual half peak-to-trough amplitude against a permuted null-distribution of amplitudes). The *gray dotted line* marks the identity line. (**B**) Same as in panel **A**, but for width of serial dependence tuning (i.e. full width at half maximum [FWHM]). (**C**) Same as in panel **A**, but relying on a model-free measure of serial dependence, allowing for the inclusion of the entire sample of subjects. SD = serial dependence.

To further quantify this consistency, we next directly correlated estimated serial dependence magnitude (i.e. half peak-to-trough amplitude of best-fitting DvM-function) and width (i.e. FWHM derived from best-fitting DvM-function) between experimental sessions for that subset of participants with significant fits for both sessions (i.e. study 1 vs. study 2: *n*: 14 vs. 13). This revealed a positive relationship for the amplitude parameter (Pearson *r* = 0.557, *p* = 0.003; Spearman *⍴* = 0.442, *p* = 0.022; BF_10_ = 17.99; Figure 7A) and evidence for no relationship for the FWHM (Pearson *r* = 0.024, *p* = 0.922; Spearman *⍴* = −0.074, *p* = 0.765; BF_10_ = 0.285; Figure 7B).

Critically, the positive relationship between the magnitudes of the single-subject serial dependence in session 1 and session 2 was not driven by those participants with the largest effects: If the amplitudes of the DvM-derived serial dependence were exactly identical in session 1 and session 2, then all data in a correlation scatterplot (see Figure 7) would fall onto the identity line (i.e. where x = y). Expressed in polar coordinates, this would mean that each data point would have an angle of exactly 45 degrees. To assess whether those participants with less strong serial dependence were, on average, equally far (or close) from the identity line than those participants with strong serial dependence, we first computed the polar angle of each observer's data (i.e. np.arctan [amplitude in session 2/amplitude in Session 1]) and then performed a median split based on average absolute serial dependence magnitude across the two sessions. We found no evidence for the split-halves to differ significantly from each other in terms of their polar coordinates (bottom half: *M* ± *SE* = 19.94 degrees ± 11.73 degrees, top half: *M* ± *SE* = 20.03 degrees ± 10.85 degrees, *t*(24) = −0.006, *p* = 0.995, Cohen's *d* = −0.002, BF_10_ = 0.363).

Together, these results demonstrate that, even in experimental settings with multiple tasks of varying predictability, only the strength (but not the tuning) of serial dependence between sessions is highly stable within a given individual.

### Between-session stability of serial dependence magnitude is invariant to operationalization of serial dependence

Up until now, we have demonstrated the between-session stability of the strength of serial dependence for a subset of our observers. Although this subselection was necessary to prevent contamination of analyses from participants with spurious DvM fits, one might argue that it is too restrictive to allow for general conclusions about the stable or fickle nature of stimulus-history effects.

To address this issue, we reran the between-session correlation with a model-free measure of serial dependence (Gallagher & Benton, 2022; Samaha et al., 2019). In contrast to the DvM-derived measures of serial dependence, this model-free measure does not rely on any assumptions and, as such, allows us to include all observers in our analysis. At the same time, it only provides an estimate of the magnitude of history biases, without providing any information about the width of the tuning.

Model-free derived single-subject amplitudes of serial dependence followed a similar pattern as model-derived estimates (see Figure 4C, Supplementary Figure S3). Indeed, there was a strong positive correlation between across-sessions serial dependence amplitudes as inferred by DvM-fitting and the model-free approach (Pearson *r* = 0.862, Spearman *⍴* = 0.795, both *p* values < 0.001; BF_10_ > 1000), confirming that both measures capture similar facets of stimulus-history effects. Critically, when relating the magnitude of single-subject model-free serial dependence in session 1 to that of session 2, we once again observed a positive association (Pearson *r* = 0.495, *p* < 0.001; Spearman *⍴* = 0.381, *p* = 0.015; BF_10_ = 36.29; Figure 7C). This positive relationship persisted even when changing the angular distance ranges to either −45 and 45 or −60 and 60. Again, as revealed by the split-half analysis, the positive relationship between the magnitudes of single-subject serial dependence in session 1 and session 2 were not driven by those participants with the largest effects (independent *t*-test between the bottom half vs. the top half: *t*(38) = −0.983, *p* = 0.332, Cohen's *d* = −0.311, BF_10_ = 0.453). The between-session stability of the strength with which stimulus history biases current behavior was not only robust to variations in the operational definition of serial dependence, but, importantly, also applied to the full sample of participants. There may thus be a unique, subject-specific phenotype of history biases.

## Discussion

Previous experience and, in particular, stimulus history shapes current perception. In hysteresis (Kleinschmidt et al., 2002; Schwiedrzik et al., 2014, 2018) or serial dependence (Fischer & Whitney, 2014), this equates to the past exerting an attractive effect, effectively rendering current percepts more similar to previous ones. A compelling hypothesis about the function which this stimulus-history effect may serve is that it may promote perceptual stability and continuity of experience in the face of noisy and, sometimes even disrupted, sensory input (Cicchini et al., 2018; Fischer & Whitney, 2014; Kiyonaga, Scimeca, et al., 2017; Kleinschmidt et al., 2002; Snyder et al., 2015). If this is indeed the case, then serial dependence should be observed in almost all circumstances (no matter their complexity), and, more importantly, given a specific setting, it should also be fairly stable in individual observers from one occasion to the next. Any interindividual variability could then be the result of differences in how much weight individuals attribute to sensory evidence versus prior information.

Whereas previous work has extensively documented the variety of stimulus types which may induce serial dependence (Alexi, Cleary, Dommisse, Palermo, Kloth, et al., 2018; Bliss et al., 2017; Fischer et al., 2020; Fornaciai & Park, 2018; Frund et al., 2014; Liberman, Manassi, & Whitney, 2018; Motala, Zhang, & Alais, 2020), much less is known about the range of tasks that elicit serial dependence. Will it persist in scenarios, in which a given stimulus requires more than one type of response? Will this also be the case if task sequence, and thus, global task structure is entirely unpredictable? Moreover, and most critically in light of the proposed function of serial dependence and related mechanisms, will the recently reported observation of high test-retest reliability of the magnitude of serial dependence (Kondo et al., 2022) and perceptual hysteresis (Van Geert et al., 2022) also hold in the context of such volatile environments, thereby providing additional evidence in favor of a stable perceptual phenotype with which the recent past shapes current perception and behavior?

Here, we answer all three questions in the affirmative: Having designed an experiment, in which participants alternated between one of two tasks (i.e. no-rotation vs. rotation) in a predictable (study 1) or unpredictable (study 2) fashion, at the group-level, we observed strong attractive serial dependence in both cases. Whereas this group-level effect was representative of the majority of individual-subject patterns, there was considerable between-subject variability, with some participants also showing a repulsion and others no influence of stimulus history on current perception. Critically, within a given observer, the magnitude of the stimulus-history effect remained stable between experimental sessions.

As such, our findings add to a sizable body of literature documenting the generalizability of serial dependence across different stimulus and task conditions (by demonstrating its existence even under global task complexity of varying predictability; Bliss et al., 2017; Cicchini et al., 2014; Cicchini et al., 2017; Corbett et al., 2011; Fischer & Whitney, 2014; Fornaciai & Park, 2018; Fritsche et al., 2017; Kok et al., 2017; Liberman et al., 2014; Manassi et al., 2021; Manassi, Liberman, et al., 2018; Taubert et al., 2016; Xia et al., 2016) and extend recent reports of its intra-observer stability (Kondo et al., 2022; Van Geert et al., 2022). They thus constitute an important piece of evidence in favor of serial dependence and related mechanisms being a general-purpose stabilizing mechanism of the visual system with a unique and stable subject-specific fingerprint.

### Serial dependence persists even in globally variable task environments

Group-level, attractive serial dependence exists for a wide range of low- and high-level features and stimulus categories, including color (Barbosa & Compte, 2020), motion direction (Alais et al., 2017; Czoschke et al., 2019; C. Fischer et al., 2020; Moon & Kwon, 2022), orientation (Cicchini et al., 2021; Collins, 2019; Fischer & Whitney, 2014; Fritsche et al., 2017; Murai & Whitney, 2021; Pascucci et al., 2019), face perception (Liberman et al., 2014; Taubert et al., 2016), interpretation of causality (Deodato & Melcher, 2022), monetary value (Morimoto & Makioka, 2022), or numerosity perception (Cicchini et al., 2014; Corbett et al., 2011; Fornaciai & Park, 2018; Fornaciai & Park, 2022). This ubiquity in feature- and/or stimulus-space is consistent with the idea that serial dependence may be a quasi-automatic general-purpose mechanism of the brain to stabilize perception and promote continuity of experience (Cicchini et al., 2018; Fischer & Whitney, 2014; Kiyonaga, Scimeca, et al., 2017; Kleinschmidt et al., 2002; Snyder et al., 2015). Our data extend and further corroborate these findings by demonstrating that, at the group- and single-subject levels, serial dependence also persists in the context of globally variable environments with multiple tasks (study 1 and study 2) and unpredictable structure (study 2; see Figures 3, 4).

Understanding the specific circumstances in which stimulus-history effects, such as serial dependence, occur is critical in order to distill their underlying functions and mechanisms. In light of the generally auto-correlated nature of the physical world, a key assumption of the hypothesized role of serial dependence is that, within certain bounds, it should be observed in many environments. Context effects, however, play a prominent role in vision and memory. Expectations built over a series of trials, for instance, lower the threshold for conscious perception (Melloni, Schwiedrzik, Muller, Rodriguez, & Singer, 2011) and coding in working memory may be task- (i.e. context-) dependent (Printzlau, Myers, Muhle-Karbe, Manohar, & Stokes, 2019). Indeed, emerging evidence suggests that the strength of serial dependence itself may be modulated by local context information (Fischer et al., 2020) and local context transitions (Kiyonaga, Manassi, et al., 2017).

Embedding our trials of interest (i.e. no-switch, no-rotation trials) in a global structure of predictable (study 1) or unpredictable (study 2) task changes could therefore have affected the strength and/or existence of serial dependence: If the past no longer serves as a reliable predictor of the future because the task requires a multitude of actions in a predictable or unpredictable fashion, then even during stretches of relative stability (i.e. several trials of the same kind repeated one after the other), the influence of the recent past on current perception and behavior could have been weakened or even entirely absent. However, despite this added level of global task complexity, we not only observed reliable serial dependence at the group- and single-subject levels in both studies, but also found its magnitude to be similar in strength when compared to previous research (e.g. Fritsche et al., 2017).

These findings are important because they support the view that serial dependence and related mechanisms may indeed be a semi-automatic, general process of the visual system to stabilize perception and promote experiential continuity. Although transitions from one task to the next might modulate serial dependence (akin to the modulations already observed in the context of stimulus transitions), according to the functional hypothesis of serial dependence, the visual system should still be biased to exploit environmental regularities if meaningful to do so. An exciting topic for future investigations will therefore be to explore exactly how such global task complexity may interact with local task complexity (e.g. at task transitions) and affect trial-wise serial dependence.

### The role of task predictability for serial dependence requires further investigation

According to a Bayesian account of perception, environmental uncertainty should affect the relative weighting of current and prior sensory evidence (De Lange, Heilbron, & Kok, 2018; Knill & Pouget, 2004). For instance, if current sensory input is ambiguous, it is beneficial to rely on previous information to guide current perception and behavior, whereas, if previous sensory evidence were ambiguous, integrating prior with current evidence would be less advantageous. Similarly, if task structure is predictable (as in study 1), then one might expect stronger reliance on information from the recent past, and, thus, stronger serial dependence than if task structure is unpredictable (as in study 2).

So far, support in favor of a fully fledged Bayesian account of serial dependence has been mixed. Although there is considerable evidence that stimulus uncertainty and confidence in one's perceptual decision modulates group-level serial dependence (Cicchini et al., 2018; Gallagher & Benton, 2022; Samaha et al., 2019; Suárez-Pinilla, Seth, & Roseboom, 2018; Van Bergen & Jehee, 2019), the observed modulation is not always in the expected direction, such that, for instance, increased uncertainty of the recent past does not reduce the strength of serial dependence (Gallagher & Benton, 2022).

The attentive reader might have noticed that, at first sight, our data, too, might appear to be at odds with a Bayesian account. As shown in Figure 3, group-level serial dependence is numerically stronger in study 2 (in which task sequence was entirely unpredictable) than it is in study 1 (with a predictable task structure). However, in addition to predictability of task structure, there were further differences between the two studies that could have affected the strength of serial dependence, including the length of the delay period (Bliss et al., 2017 and the presence of feedback (Fornaciai & Park, 2022; Fulvio, Rokers, & Samaha, 2022). For instance, in light of task difficulty in study 2, we decided to present symbolic feedback at the end of each trial (cf. Methods) in order to help participants maintain an adequate template of the required rotation angle over the course of the entire experiment. As this feedback was veridical and not modulated as a function of relative orientation (with more error feedback, for example, at orientations with stronger serial dependence), this could potentially have strengthened serial dependence (Fornaciai & Park, 2022) following correct feedback and weakened it (Fulvio et al., 2022) following incorrect feedback, by adjusting the relative weights of prior and current information. As we had not designed the current experiments with the aim of directly comparing the effects of varying degrees of task predictability on serial dependence, we caution against an over-interpretation of any differences between study 1 and study 2, and instead highlight this as an important topic for future research. Indeed, our experiments demonstrate that robust and reliable stimulus-history biases may be observed at the group- and single-subject levels even for complex, variable experimental settings with multiple tasks, and, as such, pave the way for serial-dependence research with a wider array of experimental tasks.

### Stable perceptual phenotype of the magnitude with which the past shapes present perception in individual observers in complex environments

Prominent theories, such as the Bayesian brain hypothesis, suggest that perceptual experience results from inferential processes, whereby sensory input (or evidence) is weighed by prior information and knowledge (Knill & Pouget, 2004). According to such a framework, interindividual differences in perception may be driven by different weights individuals attribute to either of these two components, with, critically, these subject-specific weights being stable from one occasion to the next. Indeed, there is evidence for certain pathologies, such as schizophrenia or autism, to shift the relative weight placed on either priors or sensory evidence (Lieder, Adam, Frenkel, Jaffe-Daz, Sahani, & Ahissar, 2019; Sterzer, Adams, Fletcher, Frith, Lawrie et al., 2018; van Leeuwen, Sauer, Jurjut, Wibral, Uhlhass et al., 2021).

Similar mechanisms could also be at play in neurotypical individuals. For instance, it has recently been suggested that a Bayesian and efficient observer model may explain concurrent attractive and repulsive history biases observed at the group-level (Fritsche et al., 2020). A critical step forward in further testing this hypothesis requires to measure (and report) serial dependence strength and tuning at the single-subject level.

Aggregating our data from the two studies, this is exactly what we set out to do in a novel experimental context. First, in line with previous reports (Bliss et al., 2017; Van Geert et al., 2022; Zhang & Alais, 2020), as indicated by both the DvM and the model-free measure, we observed large intersubject variability in the strength and tuning of the stimulus-history effects (see Figures 4, 5, Supplementary Figure S3): Whereas the majority of participants displayed attractive serial dependence with variable strength, a sizable minority had either repulsive or no discernable effects.

Critically, replicating and extending two recent reports (Kondo et al., 2022; Van Geert et al., 2022), we then provide further evidence in favor of a stable perceptual phenotype (see Figures 6, 7). Despite these considerable interindividual differences, the magnitude (but not the tuning) of the effect was stable between experimental sessions within a given individual. In contrast to the previous work, this within-subject stability of serial dependence strength arose in the context of a complex experimental structure with multiple tasks of varying predictability. This is an important finding, as it not only further qualifies the observed effect to be specific to strength (but not tuning), but also demonstrates that the high test-retest reliability of serial dependence and perceptual hysteresis as observed by Kondo and colleagues (2022) and Van Geert and colleagues (2022) is not just a particularity of the specific experimental task, but able to generalize to different contexts – an important prerequisite to count as a genuine perceptual phenotype. What is more, going one step further, we also show that this relationship was not just driven by those observers with the largest effects. Instead, as revealed by the split-half analysis, even those participants with weak or no discernable influence of stimulus history appear to have had a stable bias (or, at least, pattern of responding) between session 1 and session 2. Again, this constitutes an important, previously unexplored, piece of evidence suggesting that, going beyond serial dependence, to what extent an observer relies on the past to guide current perception has a stable phenotype.

An important question for future research to tackle will be to unravel the exact source of these interindividual differences. As stated before, a key prediction of the Bayesian brain hypothesis is that between-observer variability might arise due to differences in precision weighting of sensory evidence and prior knowledge. Perhaps due to cultural, developmental, or genetic differences, different individuals might attribute different weights to current sensory evidence versus longer-term history priors (see Lieder et al., 2019), or they might differ in the emphasis they place on stimulus history with different timescales (see Fritsche et al., 2020). To a certain extent, such perceptual phenotypes might also be adaptive and affected by learning. Indeed, there was no evidence for a between-session correlation of the tuning width of the attractive and repulsive effects in our study. Depending on the specific context, a particular observer's visual system might thus sharpen or broaden the range over which the influence of previous experience acts. However, for as long as these perceptual phenotypes are fairly stable within a given context, the observed intersubject variability could be leveraged to narrow down and, potentially even identify, the neural mechanisms subserving serial dependence and related effects.

## Conclusion

It has long been known that vision is an active process, with the brain combining noisy sensory signals with prior knowledge and information. Here, in two experiments, we manipulated global task complexity and predictability to provide novel evidence that, whereas individuals differ considerably in how stimulus history shapes their current perception, even in this variable task environment, there is a stable within-observer pattern of stimulus-history effects. Together with previous reports (Kondo et al., 2022; Van Geert et al., 2022), we propose that there may in fact be a stable perceptual phenotype of the extent with which the past influences current perception. If true, then, in line with a Bayesian framework, individual differences in perception might be explained by different weights individuals attribute to sensory evidence versus priors based on stimulus history, and these stable individual differences might be key to unravel the neural mechanism behind serial dependence.

## Supplementary Material

Supplement 1

Supplement 2

Supplement 3
