# Supplementary material for: Stable perceptual phenotype of the magnitude of history biases even in the face of global task complexity
Source: J Vis. 2023 Aug 2;23(8):4. doi: 10.1167/jov.23.8.4 (PMC10405861; doi:10.1167/jov.23.8.4)
Supplement: Supplement 1 [file jovi-23-8-4_s001.pdf]

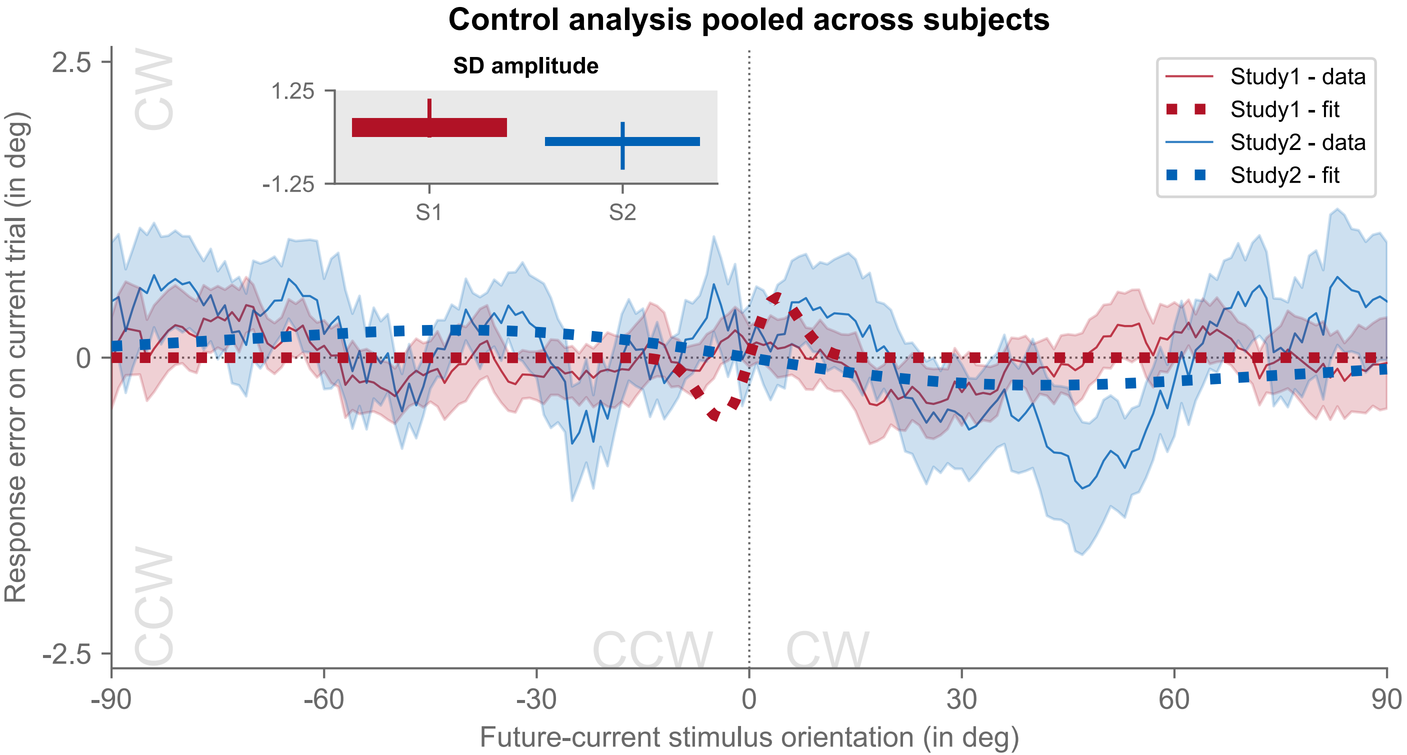

Supplementary Figure 1. Relative orientation difference between current and upcoming stimulus does not modulate response errors. Response errors on the adjustment task (i.e., response orientation - stimulus orientation; y-axis) on the current trial are shown as a function of angular distances between upcoming and current stimulus orientations (x-axis) for Study 1 (red) and Study 2 (blue). For positive y-values, the current response error was in the clockwise direction, and for positive x-values, the future stimulus was oriented more clockwise than the current stimulus. Unlike for the dependence with stimulus history, there was no systematic bias of smoothed (for visualization purposes only) mean response errors (thin colored lines with standard error [SEM] shown as shaded area) towards the upcoming stimulus. Nevertheless, we fit a derivative-of-von-Mises (DvM) function (shown as bold colored lines). Dotted lines indicate a non-significant fit with a p-value of .101 for Study 1 and .412 for Study 2 comparing actual half peak-to-trough amplitude against a permuted null-distribution of amplitudes. For consistency with Figure 3, we plot the amplitude alongside the bootstrapped standard deviations as an inset. S1 = study 1, S2 = study 2, SD = serial dependence.
