# Supplementary material for: Stable perceptual phenotype of the magnitude of history biases even in the face of global task complexity
Source: J Vis. 2023 Aug 2;23(8):4. doi: 10.1167/jov.23.8.4 (PMC10405861; doi:10.1167/jov.23.8.4)
Supplement: Supplement 2 [file jovi-23-8-4_s002.pdf]

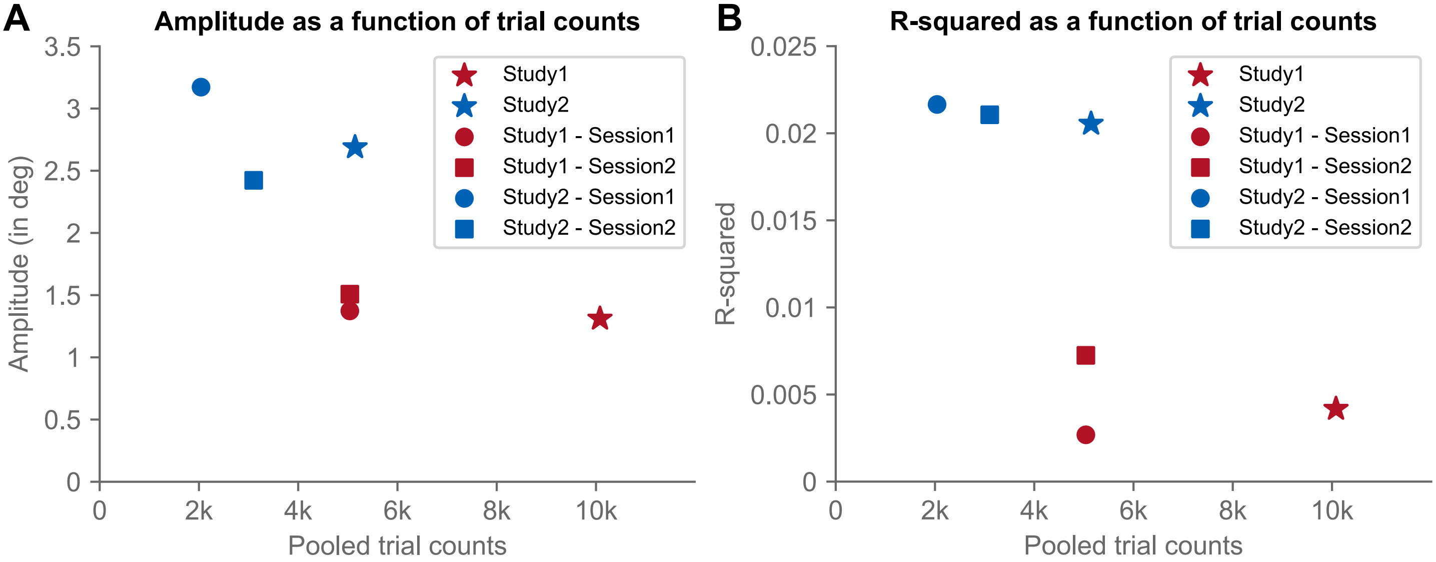

Supplementary Figure 2. Strength and quality of serial dependence does not increase with trial counts. **(A)** Half peak-to-trough amplitude as derived from the best-fitting derivative-of-von-Mises (DvM) function to pooled response errors are shown as a function of trial counts included in the model fits, separately for Study 1 (red) and Study (2), and Session 1 (circle) and Session 2 (square). **(B)** Same as in (A), but for quality of model fits as assessed by R2.
