# Supplementary material for: Stable perceptual phenotype of the magnitude of history biases even in the face of global task complexity
Source: J Vis. 2023 Aug 2;23(8):4. doi: 10.1167/jov.23.8.4 (PMC10405861; doi:10.1167/jov.23.8.4)
Supplement: Supplement 3 [file jovi-23-8-4_s003.pdf]

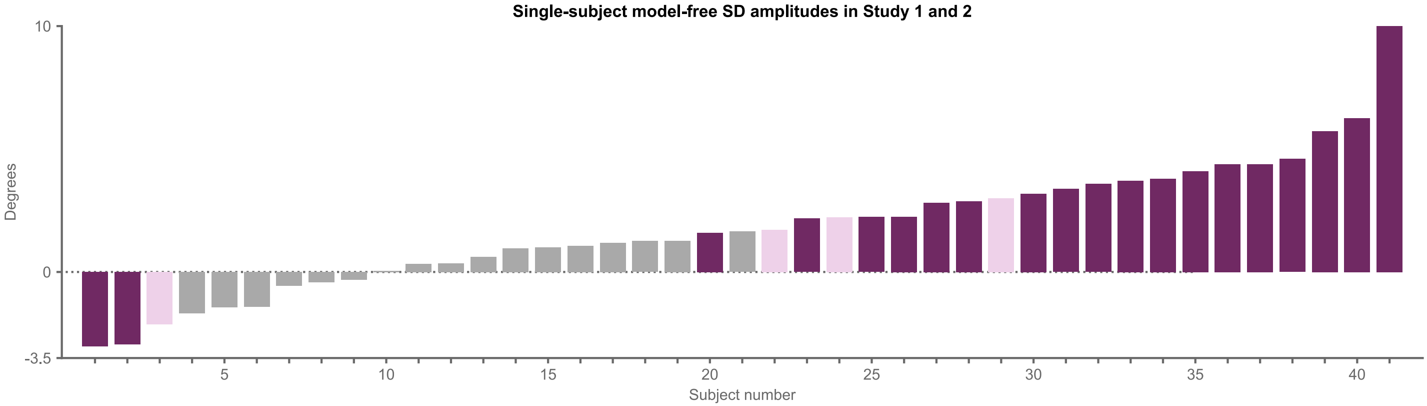

Supplementary Figure 3. Model-free derived single-subject amplitudes of serial dependence. Sorted magnitudes of model-free derived serial dependence observed for individual subjects in Study 1 and Study 2. Each bar plot shows the model-free measure of serial dependence strength of a given subject. Subjects with significant amplitudes (i.e., p < .05) are colored in dark purple, while those with amplitudes trending towards significance (i.e., p < .155) are shown in lighter colors. SD = serial dependence.
